# The Fordyce Barker Imbroglio

**Published:** 1885-02-20

**Authors:** 


### EDITORIALS AND MISCELLANEOUS.

### THE FORDYCE BARKER IMBROGLIO.

In the Medical Record, of New York, for January 10, we have statements from both sides of this issue, from which disinterested parties will infer that, in the signature-book of the New York Academy of Medicine, Dr. Fordyce Barker enrolled his name on June 4, 1854, as a graduate of Paris in 1844, and that he made oath, on the 4th day of October, 1880, in the office of the Commissioner of Deeds, New York City, that in the year 1844 he received a diploma granted by authority of the Ecole de Medicine, Paris, France; and that, by authority of said diploma, he has been practicing medicine since the year 1844. By his own statements, as reported by Andrew H. Smith, M. D., chairman of the committee on Ethics, Dr. Fordyce Barker secured, in 1861, his diploma from the school of medicine in Paris, dated in 1845—of which he could not have known previously, so as to have authorized the entry made in 1854, nor of which he could have availed himself as authority for practicing medicine since the year 1844 That Dr. Barker exhibited a diploma, purporting to be from Paris, upon his return to New York, in 1861, does not in any manner affect the issue raised upon his not having such diploma at a prior date; and he does not claim to have had any knowledge of it until the occasion, in 1861, on which he disclaimed the title of being a graduate of Paris when dining in Paris with M. Trousseau, in company with the Minister of Public Instruction. This is the whole matter; and the medical public will decide impartially between their colleagues who presented the charges, and the committee which decided that "the charge and specifications are not sustained." *Fiat justicia, si ruit coelum.*

